# Thymic involution beyond T-cell insufficiency

**DOI:** 10.18632/oncotarget.4970

**Published:** 2015-07-21

**Authors:** Brandon Coder, Dong-Ming Su

**Affiliations:** Department of Cell Biology and Immunology, University of North Texas Health Science Center at Fort Worth, Fort Worth, TX, USA

**Keywords:** Immunology and Microbiology Section, Immune response, Immunity, thymus, T cells, aging, inflammation

It is well known that age-related involution (shrinkage) of the thymus (a central cellular immune organ) results in decreased output of naïve T cells [1]. The insufficiency of naïve T cells significantly reduces the T cell receptor repertoire diversity, thereby leading to immunosenescence. However, the deleterious effects of thymic involution extend beyond diminished output. It also increases the release of harmful autoreactive T cells which usually results in autoimmune diseases [2], e.g. the autoimmune regulator gene (*Aire*) mutation-induced autoimmune-polyendocrinopathy-candidiasis ectodermal dystrophy. However, the mechanisms and clinical implications of the aging-induced increased release of autoreactive T cells are not clear, especially since not all elderly individuals develop specific autoimmune diseases.

Most aged individuals possess low-grade, but above baseline, sustained pro-inflammatory factors, such as interleukin-6 and -1, tumor necrosis factor alpha, and C-reactive protein. This chronic inflammatory state, associated with the aging process termed “inflammaging”, has been implicated in the poor prognosis of virtually every age-related disease. Studies have shown that inflammaging can be attributed to senescent cell-induced “senescence-associated secretory phenotype” [3], and the progressive activation of immune cells, mainly elicited by latent viral infections (“foreign-reaction”) [4]. A recent study has demonstrated that the increased release of autoreactive T cells from the involuted thymus also contributes to the development of inflammaging via an “self/auto-reaction” [5]. Not only has this study revealed a new source of inflammaging, but it has also demonstrated that the age-related increased release of autoreactive T cells is clinically significant, despite the lack of an overt autoimmune disease.

The study [5] focuses on age-related thymic involution and thymic function in the context of establishment of immune tolerance, which is mainly accomplished through two mechanisms: thymocyte negative selection and the generation of thymic CD4^+^FoxP3^+^ T regulatory cells (Tregs). Firstly, using a *FoxN1* conditional knockout (cKO) mouse model of accelerated thymic involution [6] crossed to a *Rag2*-GFP reporter mouse (to track newly released T cells, termed recent thymic emigrants, RTEs), it was demonstrated that RTEs derived from the involuted thymus adopt an autoreactive phenotype. Generally, RTEs are not in a highly active state because they have not yet encountered their cognate antigens. The increased activation of CD4^+^ and CD8^+^ RTEs from the involuted thymus in the absence of infection suggests that these RTEs are autoreactive T cells that are responding to peripheral self-antigens. To confirm this, an IRBP (interstitial retinol-binding protein) immunization model was used to amplify and detect autoreactive T cell clones within a polyclonal T cell repertoire. Mice with an involuted thymus showed a significant expansion of activated IRBP specific T cells, while they were mostly undetectable in littermate controls with a normal thymus.

It is well-known that most autoreactive T clones are eliminated through thymocyte negative selection. Therefore, the question arises as to why so many autoreactive T clones are released from the involuted thymus. Negative selection in the involuted thymus was then examined using a triple genetically engineered mouse model. This was done by transplanting a newborn mouse thymic lobe from a RIP-mOVA^+^ and *FoxN1*-cKO background into the kidney capsule of young adult OT-II TCR-transgenic mice. RIP-mOVA is a transgene, which expresses chicken ovalbumin as a neo-self-antigen in an Aire-dependent manner in the thymic medulla where negative selection takes place. The OT-II TCR transgenic mice have CD4^+^ thymocytes that specifically recognize RIP-mOVA. Once these OT-II TCR transgenic thymocytes encounter RIP-mOVA^+^ medullary thymic epithelial cells (mTECs), they are signaled to undergo exaggerated negative selection. However, when bone marrow progenitor cells from OT-II TCR transgenic hosts were seeded into the grafted RIP-mOVA^+^/*FoxN1*-cKO thymus in the kidney capsule, the exaggerated negative selection was not observed. Furthermore, mTECs from *FoxN1-*cKO mice were found to have diminished expression of Aire, suggesting that impaired clonal deletion likely results from defects related to Aire-dependent promiscuous gene expression of tissue-specific self-antigens. This finding confirmed that negative selection is impaired in the involuted thymus.

In a healthy individual, the escape of a few autoreactive T cells from negative selection is not necessarily an issue because thymus-derived Tregs are able to suppress the autoimmune response [7]. The study went on to ask whether thymic involution also disrupts Treg generation [5]. Treg generation and function were then analyzed. The results showed that thymic involution does not impair Treg generation, but may even enhance it. Peripheral Tregs from the involuted thymus were demonstrated to possess adequate suppressive capabilities. It is known that in naturally aged mice Tregs accumulate in the periphery and inflammatory cell infiltration into non-lymphoid tissues is elevated. To test whether these phenomena result from intrinsic defects related to thymic involution or are controlled by extrinsic features of the aged peripheral microenvironment, naturally aged spleen cells were transplanted into young *Rag* gene knockout mice, which do not have endogenous T and B cells. The age-related Treg accumulation and enhanced survival via diminished expression of the pro-apoptotic *Bim* gene in the Tregs could be reversed by the young microenvironment, while the inflammatory cell infiltration into non-lymphoid tissues could not. These data imply that changes in peripheral aged Tregs are non-intrinsic, while inflammatory infiltration is driven by intrinsic defects related to perturbed negative selection.

Together, these findings demonstrate that age-related thymic involution is involved not only in immunosenescence associated with the insufficient output of naïve T cells, but also in the emergence of inflammaging via the increased release of autoreactive T cells. Therefore, therapeutically targeting thymic involution should present a promising strategy for attenuating chronic inflammation, thereby reducing a major risk factor associated with morbidity and mortality in virtually every chronic age-related disease.
